# Assessment of the stiffness of the upper trapezius muscle in a group of asymptomatic people with cervical spine rotation asymmetry

**DOI:** 10.1371/journal.pone.0298544

**Published:** 2024-02-22

**Authors:** Michał Wendt, Małgorzata Waszak

**Affiliations:** Department of Medical Biology, Poznan University of Physical Education, Poznań, Poland; Universidad Complutense de Madrid Escuela Universitaria de Enfermeria Fisioterapia y Podologia, SPAIN

## Abstract

This study investigated the relationship between the stiffness of the upper trapezius muscle and the range of rotational movement of the cervical spine. A total of 60 right-handed asymptomatic students participated in the study. Participants (N = 22) characterised by asymmetry in rotational movements were selected for the experimental group. A difference of ≥10° between right and left rotation of the cervical spine was considered asymmetrical. The control group (N = 38) included participants whose rotation difference was < 10°. Belonging to the experimental or control group did not significantly differentiate trapezius muscle stiffness. The rotation side differentiated the stiffness of the right and left trapezius muscles only in the group of people with rotational movement asymmetry. There were high correlation coefficients between right cervical rotation and the stiffness of the muscle on the right side, and between rotation to the left and the stiffness of the muscle on the left side. There is a relationship between the stiffness of the right and left upper trapezius muscles and the range of right and left rotational motion of the cervical spine. Stiffness of the upper trapezius correlates more strongly with rotation to the side on which the muscle lies than to the opposite side. Increased stiffness of the upper trapezius muscle on the side of limited cervical spine rotation is likely to be determined by the muscle fibre stretching mechanism.

## Introduction

In recent years, research related to the assessment of biophysical parameters and their relationship with dysfunctions of the musculoskeletal system in many social groups exposed to various types of stress on muscles, tendons and joints has become more and more popular. For this purpose, researchers are increasingly using MyotonPRO. It is a relatively new hand-held device that allows characterising the biomechanical properties of superficial soft tissues in a simple and non-invasive way [1]. It works by applying a mechanical impulse to the skin, which is then transmitted to the underlying soft tissue and muscle [2]. The device allows you to test tone, stiffness and flexibility [3]. MyotonPRO can also measure other parameters of muscles, such as creep and viscoelastic stress relaxation [4].

Stiffness refers to a body that resists deformation when changing its length [5]. In the scientific literature describing the viscoelastic properties of skeletal muscles, the term passive stiffness is used. It is the relationship between the length of muscle fibers and their tension at rest [6]. The value of passive stiffness of a given muscle will also depend on the range of motion in the joint and muscle strength [7]. A person’s motor skills are also important here. The measurement of this parameter is quite complex and depends on the viscoelastic properties of adjacent anatomical structures such as: joint capsules, ligaments or fascia [7]. The term dynamic stiffness is also used to characterize muscle tissue. This parameter concerns deformable bodies that store and return elastic energy [8]. The dynamic stiffness (S) is measured in N/m. This biophysical parameter reflects the muscle’s ability to resist contractions or external stresses to deformation [3]. It can also be defined as the resistance of the muscle during its passive stretching [9]. It is mainly responsible for transmission and absorption of energy during various activities of the musculoskeletal system [10]. Stiffness is an important biophysical parameter that indicates the condition of the soft tissue tested [11]. Increased stiffness correlates with exercise [12], pain [11,13], muscle dysfunction [11] and tendinopathy [14].

Active range of motion (AROM) is one of the most important functional parameters used in various research related to spine biomechanics [14]. It enables assessing the proper functioning of the cervical, thoracic and lumbar spine in a group of symptomatic or asymptomatic people [14]. A reduced angular value of AROM may result in various limitations in the performance of everyday activities [14]. Any deviations in functional parameters may consequently lead to negative structural changes [15]. In the case of the spine, degeneration and dysfunction may occur in the area of intervertebral discs, intervertebral joints, vertebrae, ligaments and muscles [15–18]. AROM in the direction of rotation is believed to be the most important range of motion for proper cervical spine functioning [18].

The trapezius muscle is a large, superficial, flat muscle lying on the dorsal side of the upper torso and neck [19]. It comprises three parts that perform different functions. The upper part of the muscle is responsible for lifting and retracting the shoulder girdle. It cooperates with the serratus anterior muscle in correctly positioning the scapula during above-horizontal arm movements. With a stabilised scapula, the upper trapezius is involved in head movements [19]. In the case of bilateral contraction, the muscle causes extension of the head, while with unilateral contraction it rotates the head in the opposite direction [19]. The scientific literature shows that the area of the shoulder girdle and the trapezius muscle itself are crucial from the point of view of various shoulder and neck overload syndromes [20].

Some muscular and joint dysfunctions also occur in asymptomatic people [21]. They are the result of the accumulation of overloads occurring during incorrect positions, posture and movement patterns, accompanying various activities of everyday life [22]. If untreated, they become more severe and have an increasingly negative effect on the locomotor system [21]. The ability to diagnose functional disorders quickly and accurately is crucial for the implementation of effective therapy. In today’s physiotherapy, diagnostic and therapeutic elements are constantly being improved through continuous scientific research assessing the correlation of various functional, structural, biophysical and subjective parameters characterising the state of the musculoskeletal system.

Despite the literature confirming the biological significance of muscle stiffness, there appears to be no focused research on whether there is a relationship between upper trapezius muscle stiffness and cervical spine mobility. Therefore, the purpose of this study was to compare upper trapezius muscle stiffness on both sides in individuals with symmetrical versus asymmetrical AROM (rotational movements) of the cervical spine.

## Material and methods

**Fig 1:**
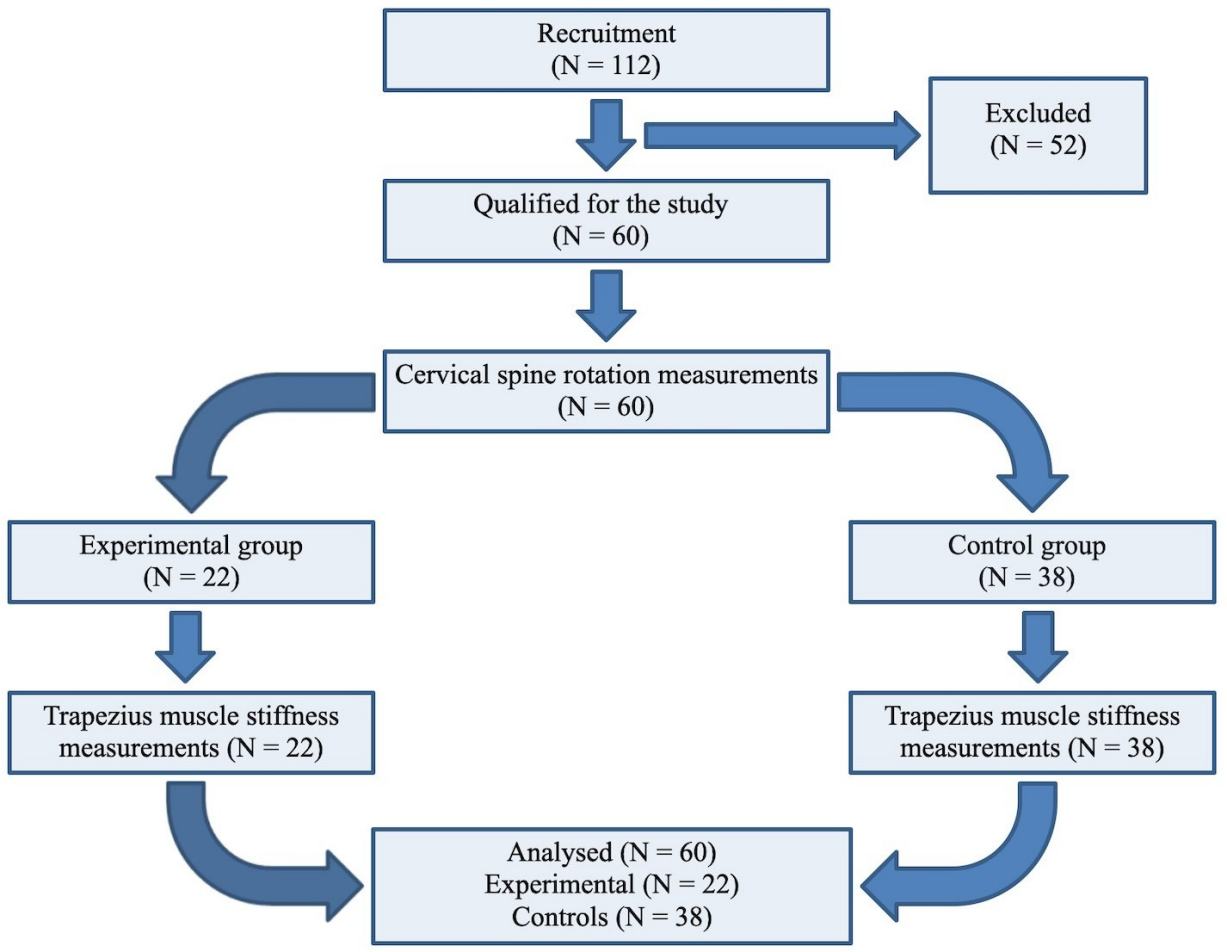
Flow of participants through all phases of a research study.

The conducted study is classified as observational (case-control). The study design was based on Equatornetwork and STROBE checklist was used [23]. All measurements were carried out in the same place: Department of Biology and Anatomy of the Poznan University of Physical Education. During the measurements in the test room, the temperature was constant (23°C). The room was ventilated. Recruitment for this study began on April 13, 2020 and ended on May 4, 2020. A detailed description of the flow of participants through the individual phases of the study is presented in Fig 1.

The following efforts were made to prevent potential sources of bias: fidelity to the protocol, avoidance of unintended interventions or co-interventions, patient blinding, outcome assessor blinding, data analyst blinding, appropriate statistical methods, accounting for dropouts, obtaining complete data, complete reporting of all prespecified outcomes.

### Participants

A total of 60 right-handed first year students participated in the study. These students practiced symmetrical sports (swimming, running, cycling, gym, roller-skating) as amateurs. All participants were tested for active rotation movements in the cervical spine. Two research groups were included in the study, depending on the angular values of the tested mobility. The experimental group (E; N = 22) comprised participants characterised by asymmetry in rotational movements. A difference of ≥10° (in absolute value) between right and left rotation of the cervical spine was considered asymmetrical. The control group (C; N = 38) included participants whose rotation difference was <10°. Both groups were divided into R1 and R2 subgroups due to the side of limited or less rotation. And so, R1 included people with limited (in the case of the control group ‐ smaller) right rotation, and in the R2 subgroup ‐ people with limited (in the case of the control group ‐ smaller) left rotation. The exclusion criteria included: age >21 years, pain in the cervical spine or shoulder girdle, any neurological symptoms in the area of the upper limb, operations in the area of the cervical spine or shoulder girdle, professional sports and practicing asymmetrical sports, scoliosis, any disorders of the physiological curvatures of the spine. A physiotherapist with over 10 years of professional experience supervised the proper course of the recruitment and diagnostics process. The characteristics of the study group are included in Table 1.

**Table 1:** Characteristics of the study participants.

Parameter	Category	All		Group E		Group C	
		N	%	N	%	N	%
Age [years]	19	8	13.3	2	10	6	15.8
	20	48	80.0	18	80	30	79.0
	21	4	6.7	2	10	2	5.2
Gender	men	36	60.0	14	63.6	22	57.9
	women	24	40.0	8	36.4	16	42.1
Weight [kg]	60–70	21	35.0	8	36.4	13	34.2
	71–80	22	36.7	10	45.5	12	31.6
	81–90	12	20.0	3	13.6	9	23.7
	91–100	5	8.3	1	4.5	4	10.5
Height [cm]	160–170	17	28.3	6	27.3	11	28.9
	171–180	30	50.0	11	50.0	19	50.0
	181–190	13	21.7	5	22.7	8	21.1
BMI [kg/m^2^]	17–18.5	0	0	0	0	0	0
	18.5–25	60	100	22	100	38	100
	25–30	0	0	0	0	0	0
Physical activity	1x a week	8	13.3	3	13.6	5	13.2
	2x a week	45	75.0	17	77.3	28	73.7
	3x a week	7	11.7	2	9.1	5	13.1

### Measurement methods

**Fig 2:**
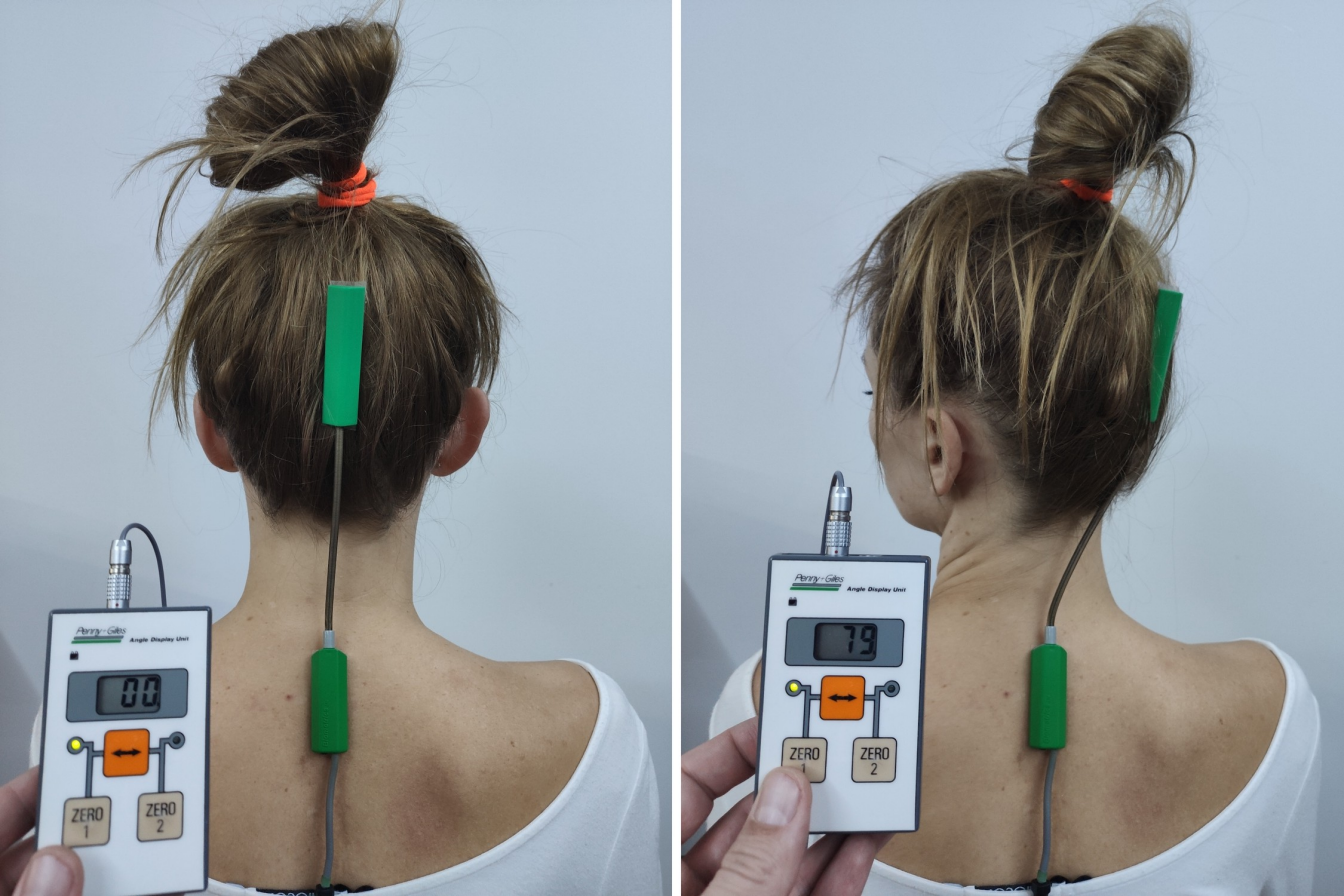
Goniometric measurements of the cervical spine rotation in the starting position (left photo) and end range position (right photo).

To perform the required angular measurements of the spine’s mobility, a Penny & Giles strain gauge electrogoniometer was used together with a Q110 single-plane sensor (Fig 2). The sensors were placed in the following anatomical regions: external occipital tuberosity (the lower edge of the upper sensor) and the spinous process of C7 (the upper edge of the lower sensor). The study assessed the angular values of the rotation of the cervical spine in both directions. Goniometric measurements were made in a sitting position. The measurement methodology according to Lewandowski [24] was used. Double-sided tape (Biometrics) was used to attach the sensors to the participant’s body. The result of the angular range of rotation (for each side) was the mean of three measurements performed directly in succession. The Penny & Giles strain gauge electrogoniometer is a reliable and repeatable measurement tool used to measure the segmental mobility of the spine [24].

**Fig 3:**
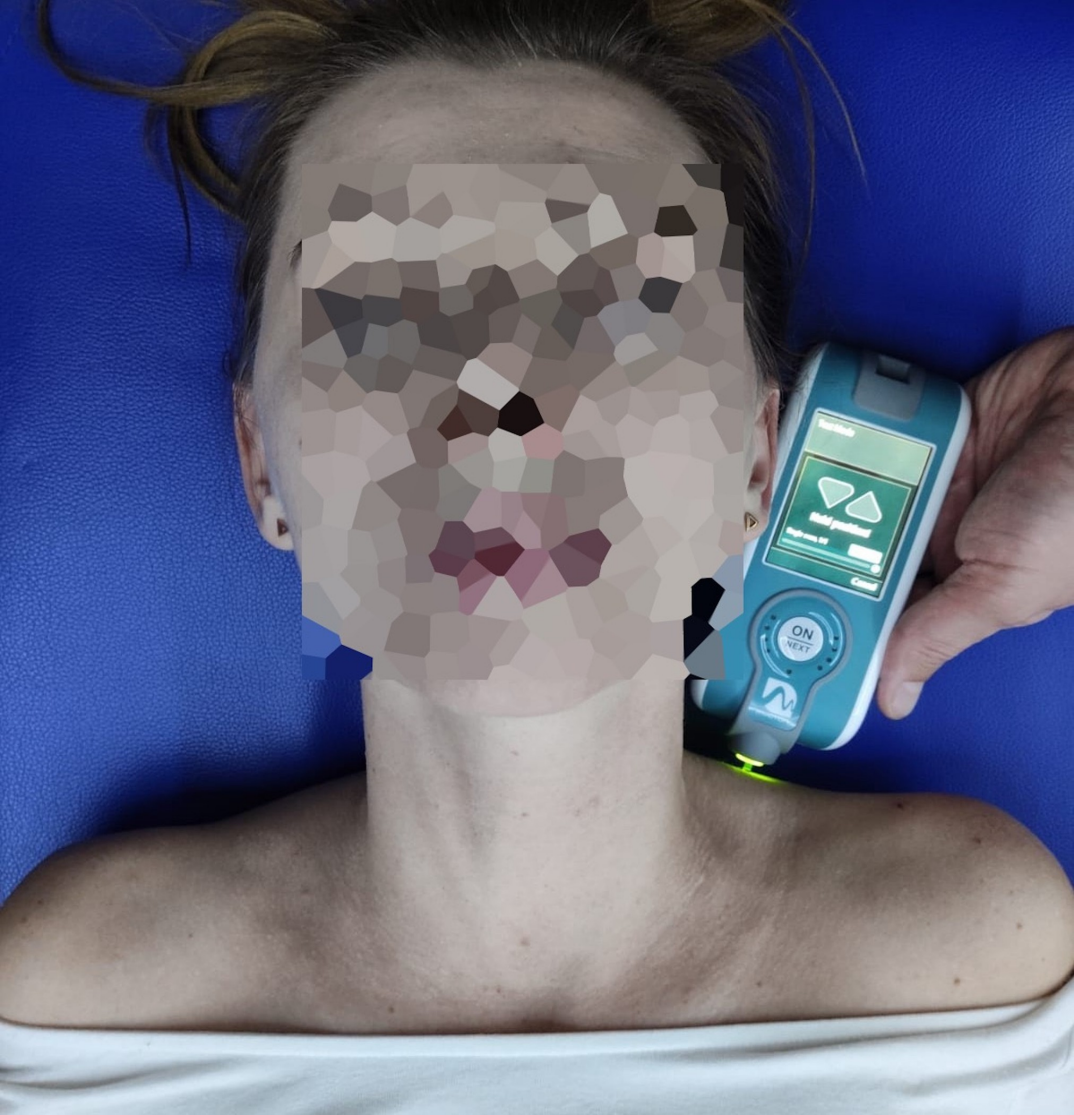
Stiffness measurement of the left upper trapezius muscle using MyotonPRO.

To assess the dynamic stiffness (S, measured in N/m) of the upper trapezius muscle, the MyotonPRO myotonometer by Myoton Muscle Diagnostics was used (Fig 3). Measurements were made on the upper trapezius muscle (right and left) at a point located in the middle of the section between the spinous process of the C7 vertebra and the acromion of the scapula (anterior apex, next to the acromioclavicular joint line). The participants were in the supine position. The probe of the device was placed perpendicular to the surface of the skin covering the examined muscle. Pattern composer had the following settings: tap time, 15 ms; interval, 0.8 s; mode, multiscan (five repetitions). MyotonPRO is a reliable and repetitive tool to measure the biophysical parameters of superficial soft tissues such as skin [25], muscle [26–28] or tendon [29]. The scientific literature indicates that this also applies to the upper trapezius muscle in the context of assessing dynamic stiffness [30]. MyotonPRO is reliable to assess biomechanical properties in a healthy population [31] and in people with pathological conditions [32,33].

### Statistical methods

The sample size was determined on the basis of a previous study. Using the ’Power Analysis’ tool in Statistica 13.3 software (TIBCO Software Inc.; Palo Alto, CA, USA), the correlation coefficient (a variable calculated from previous studies), target test power and alpha value were substituted. A total of 9 cases were calculated, to yield at least 80% power of detecting an effect as statistically significant at the 0.05 α level, with a detectable effect size of 0.8.

The collected data on the range of rotational movement to the right and left as well as the stiffness of the right and left trapezius muscles were statistically analyzed in Statistica version 13.3

The normality of the distribution of the studied variables was checked with the Shapiro-Wilk test, and the equality of variances with the Levene test. To compare the stiffness values of the right and left trapesius muscles between the two groups (E and C), the Student’s t-test for independent samples or the Mann-Whitney test were calculated.

In order to determine the possible differentiation of the stiffness of the right and left trapezius muscles in the groups distinguished by: the presence of asymmetry in the area of rotational movement and the side of limited or less rotation, the analysis of variance with 4 levels (ER1, ER2, CR1, CR2) and post-hoc tests (LSD) with Bonferroni correction were applied, and Student’s t-test for independent samples or Mann-Whitney U test was calculated.

In order to investigate the effect of independent variables, i.e. right trapezius ST (R) and left ST (L) stiffness, on the dependent variables: the range of cervical right rotation (CRR) and cervical left rotation (CLR), the progressive stepwise regression was used.

### Ethics

Participation in the study was free, voluntary and anonymous. It was possible to opt out of the study at any stage. Voluntary, written informed consent of the participants and the approval of the Bioethics Committee at the Poznan University of Medical Sciences (Number: 232/20) were obtained. The experimental procedures were conducted in conformity with the Declaration of Helsinki. The relevant guidelines and regulations of the local institute were strictly followed when conducting the study.

### Research objectives and hypotheses

The main objective of the study was to answer the following question: is there a relationship between the stiffness of the upper trapezius muscle and the range of rotational movement of the cervical spine? The following research tasks aimed to answer this question:

1. determination of the differentiation of the stiffness of the right and left upper trapezius muscles depending on the occurrence of rotational movement asymmetry (differences between group E and group C) and the side of limited rotation (differences between groups ER1 and ER2 and groups CR1 and CR2);
2. determine whether there is a correlation between limited rotation and the stiffness of the upper trapezius muscle lying on the side of limited rotation and the stiffness of the upper trapezius muscle lying on the opposite side;
3. determine whether there is a correlation between the difference in right and left rotation and the difference in stiffness in the right and left upper trapezius muscle.

The following hypotheses were made:

1. There is a relationship between the stiffness of the right and left upper trapezius muscles and the range of right and left rotational motion of the cervical spine.
2. The upper trapezius muscle lying on the side of limited cervical spine rotation will be characterized by increased stiffness compared to the muscle on the opposite side.

## Results

The first research task was to determine the stiffness of the right and left trapezius muscles depending on the extent of the asymmetry of rotational movement and the side of restricted movement.

The muscle stiffness in the groups distinguished due to the presence of asymmetry in rotational movement (E and C) was determined depending on the side of the limited or less rotational movement and is presented in Table 2. Group R1 includes people who were characterized by lower right-sided rotation than left-sided rotation, while in the R2 group people characterized by lower left-sided than right-sided rotation. In the control group there were 21 people with a lower left than right rotation and 17 people with less right than left rotation, while in the experimental group 10 people with limited left rotation and 12 with limited right rotation.

**Table 2:** Basic characteristics of the stiffness of the right and left trapezius muscles in the experimental (E) and control (C) groups depending on the side of limited rotation (less right rotation–R1, less left rotation–R2).

Group	N	Right trapezius stiffness		Left trapezius stiffness	
		X	SD	X	SD
			
E	22	261.52	45.34	249.89	46.41
ER2	10	221.26	24.49	211.04	11.03
ER1	12	295.07	27.06	296.51	21.22
C	38	251.01	42.46	250.48	40.58
CR2	21	243.76	39.73	241.71	39.75
CR1	17	259.97	45.19	257.58	40.79
Overall	60	254.87	43.46	250.26	42.42

E–experimental group, ER2 ‐ experimental group with limited left rotation, ER1 ‐ experimental group with limited right rotation, C ‐ control group, CR2 ‐ control group with less left than right rotation, CR1 ‐ a control group with less right than left rotation, N–quantity, X ‐ arithmetic average, SD–standard deviation.

The stiffness of the trapezius muscle (right and left) does not significantly differentiate the experimental and control groups (Table 3).

**Table 3:** Comparison of the stiffness of the right and left trapezius muscle between the experimental group (E) and the control group (C).

Variable	X (E)	X (C)	SD E)	SD (C)	N (E)	N (C)	df	t	p
ST (R)	261.52	251.01	45.34	42.46	22	38	58	0.901	0.371392^A^
ST (L)	249.89	250.48	46.41	40.58	22	38	58	-0.051	0.959407^B^

ST (R) ‐ stiffness of the right trapezius muscle, ST (L) ‐ stiffness of the left trapezius muscle, E ‐ experimental group, C ‐ control group, $\bar{X}$ - average; SD ‐ standard deviation; t ‐ value of the Student’s t-test for independent variables or Mann-Whitney U test; p ‐ test probability, A ‐ Student’s t-test for independent variables, B ‐ Mann-Whitney U test.

To find out whether there is a difference in the stiffness of the trapezius muscle (right and left) depending on the side of limited rotation in the group of people with asymmetry (E) and less rotation in the group without rotational movement asymmetry (C), a one-way analysis of variance with 4 levels was used (Table 4). The distinguished levels of ER1, ER2, CR1, CR2 differentiate the stiffness of both the right and left trapezius muscles.

**Table 4:** Results of one-way analysis of variance with 4 levels (ER1, ER2, CR1, CR2) for right and left trapezius muscle stiffness.

Variable	Analysis of variance			
	F	df_1_	df_2_	p
ST (R)	8.1000	3	56	0.000143**
ST (L)	12.3223	3	56	0.000003**

ST (R) ‐ right muscle stiffness; ST (L)–left muscle stiffness; F ‐ statistics F; df_1_ = K– 1; df_2_ = N–K; N ‐ number of observations; K ‐ number of levels of a given factor = 4; p ‐ probability of test statistics

** significance at the level α≤0.01.

To determine which categories of upper trapezius muscle stiffness the difference is statistically significant, the LSD test was performed separately for the right and left muscles (Table 5). To counteract the problem of multiple comparisons, the Bonferroni correction was applied. This approach involved dividing the nominal significance level of each of the related tests by six. So, for α = 0.01, p < 0.0017 is required; for α = 0.05, p < 0.0083 is required.

**Table 5:** Significance of the differences between the pairs of the distinguished categories of upper trapezius muscle stiffness (ER1, ER2, CR1, CR2) ‐ LSD test.

Muscle stiffness categories		For ST (R)			
		(p) ER2	(p) ER1	(p) CR2	(p) CR1
For ST (L)	(p) ER2		0.000022**	0.121490	0.011668
	(p) ER1	0.000000**		0.000352**	0.015413
	(p) CR2	0.004038*	0.000353**		0.187795
	(p) CR1	0.000149**	0.019407	0.155720	

ST (R) ‐ stiffness of the right upper trapezius muscle, ST (L) ‐ stiffness of the left upper trapezius muscle, ER2 –experimental group with limited rotation to the left, ER1 ‐ experimental group with limited rotation to the right, CR2 –control group with less rotation to the left, CR1 ‐ control group with less rotation to the right, p ‐ test probability

** differences significant at the level of α ≤0.01 after Bonferroni correction

* differences significant at the level of α≤0.05 after the Bonferroni correction.

The LSD test showed a significant difference (α < 0.01) between categories R1 and R2 within the experimental group and between categories ER1 and CR2 for the stiffness of the right upper trapezius muscle; a significant difference (α < 0.05) between categories ER2 and CR1 (Table 5). There were similar results for the distinguished stiffness categories of the left trapezius muscle (Table 5).

It is interesting whether in the experimental group and in the control group, the stiffness of the muscle on the side of limited or less right rotation is significantly different from the stiffness of the muscle on the side of limited or less left-sided rotation. The Student’s t-test for independent variables and Mann-Whitney U test used for this purpose showed statistically significant differentiation only in people in group (E) with rotational movement asymmetry (Table 6).

**Table 6:** Comparison of the stiffness of the right and left trapezius muscles between participants with limited left-sided rotation (R2) and participants with limited right-sided rotation (R1) in the experimental group (E).

Variable	X (ER2)	X (ER1)	SD (ER2)	SD (ER1)	N (ER2)	N (ER1)	df	t	p
ST (R)	221.26	295.07	24.49	27.06	10	12	20	-6.646	0.000002**^A^
ST (L)	296.51	211.04	21.22	11.03	10	12	20	12.160	0.000000**^B^

ST (R) ‐ stiffness of the right trapezius muscle, ST (L) ‐ stiffness of the left trapezius muscle, ER2 –experimental group with limited rotation to the left, ER1 ‐ experimental group with limited rotation to the right, $\bar{X}$ - average; SD ‐ standard deviation; t ‐ value of the Student’s t-test for independent variables or Mann-Whitney U test; p ‐ test probability, A ‐ Student’s t-test for independent variables, B ‐ Mann-Whitney U test.

In the control group (C), who did not show asymmetry in rotation, people with less right-sided rotation did not differ significantly in the stiffness of the trapezius muscle (right and left) from those with less left-sided rotation (Table 7).

**Table 7:** Comparison of the stiffness of the right and left trapezius muscles between participants with less left rotation (R2) and participants with less right rotation (R1) in the control group (C) using the Student’s t-test for independent variables.

Variable	X (CR2)	X (CR1)	SD (CR2)	SD (CR1)	N (CR2)	N (CR1)	df	t	p
ST (R)	243.76	259.97	39.73	45.19	21	17	36	-1.176	0.247419
ST (L)	241.71	257.58	39.75	40.79	21	17	36	1.206	0.235805

ST (R) ‐ stiffness of the right trapezius muscle, ST (L) ‐ stiffness of the left trapezius muscle, CR2 –control group with less rotation to the left, CR1 ‐ control group with less rotation to the right, $\bar{X}$ - average; SD ‐ standard deviation; t ‐ value of the Student’s t-test for independent variables; p ‐ test probability.

In order to investigate the influence of independent variables, ie right trapezius ST (R) and left ST (L) stiffness, on the dependent variables: the range of cervical right rotation (CRR) and cervical left rotation (CLR), progressive stepwise regression method was used. The value of F = 2 was used to enter the variables into the model. In the next stage, insignificant independent variables were removed, i.e. for the variable CRR ‐ left muscle stiffness, and for the CLR variable ‐ right muscle stiffness. Based on the results of the analysis, it can be concluded that the regression model taking into account the independent variable: right muscle stiffness [N/m] allows to explain over 82% of the variance of the CRR variable [°] (Table 8), and the regression model taking into account the independent variable: left muscle stiffness [N/m] allows to explain 66% of the variance of the CLR variable [°] (Table 9). In both of these cases, the value of the F statistic and the corresponding p-test probability level confirm a statistically significant linear relationship. Moreover, the values of the t-statistic used to assess the significance of the intercept and the slope indicate that these parameters are significantly different from zero.

**Table 8:** Results of multiple regression analysis for the dependent variable CRR and independent variable ST (R).

N = 60	Summary of the regression of the dependent variable: CRR R = 0.9069 R^2^ = 0.8226 F (1.58) = 268.86 p<0.0000 Standard error of estimation: 3.2027			
	r	b	t (58)	p
y-intercept		116.530	46.987	0.0000**
right muscle stiffness ST(R) [N/m]	-0.9069	-0.157	-16.397	0.0000**

R–multiple correlation coefficient, R^2^ –multiple determination coefficient, r–correlation coefficient, b–slope.

**Table 9:** Multiple regression analysis results for the CLR dependent variable and the ST (L) independent variable.

N = 60	Summary of the regression of the dependent variable: CLR R = 0.8120 R^2^ = 0.6594 F (1.58) = 112.27 p < .0000 Standard error of estimation: 4.0239			
	b*	b	t (58)	p
y-intercept		109.797	35.034	0.0000**
left muscle stiffness [N/m]	-0.8120	-0.131	-10.596	0.0000**

R–multiple correlation coefficient, R^2^ –multiple determination coefficient, r–correlation coefficient, b–slope.

**Fig 4:**
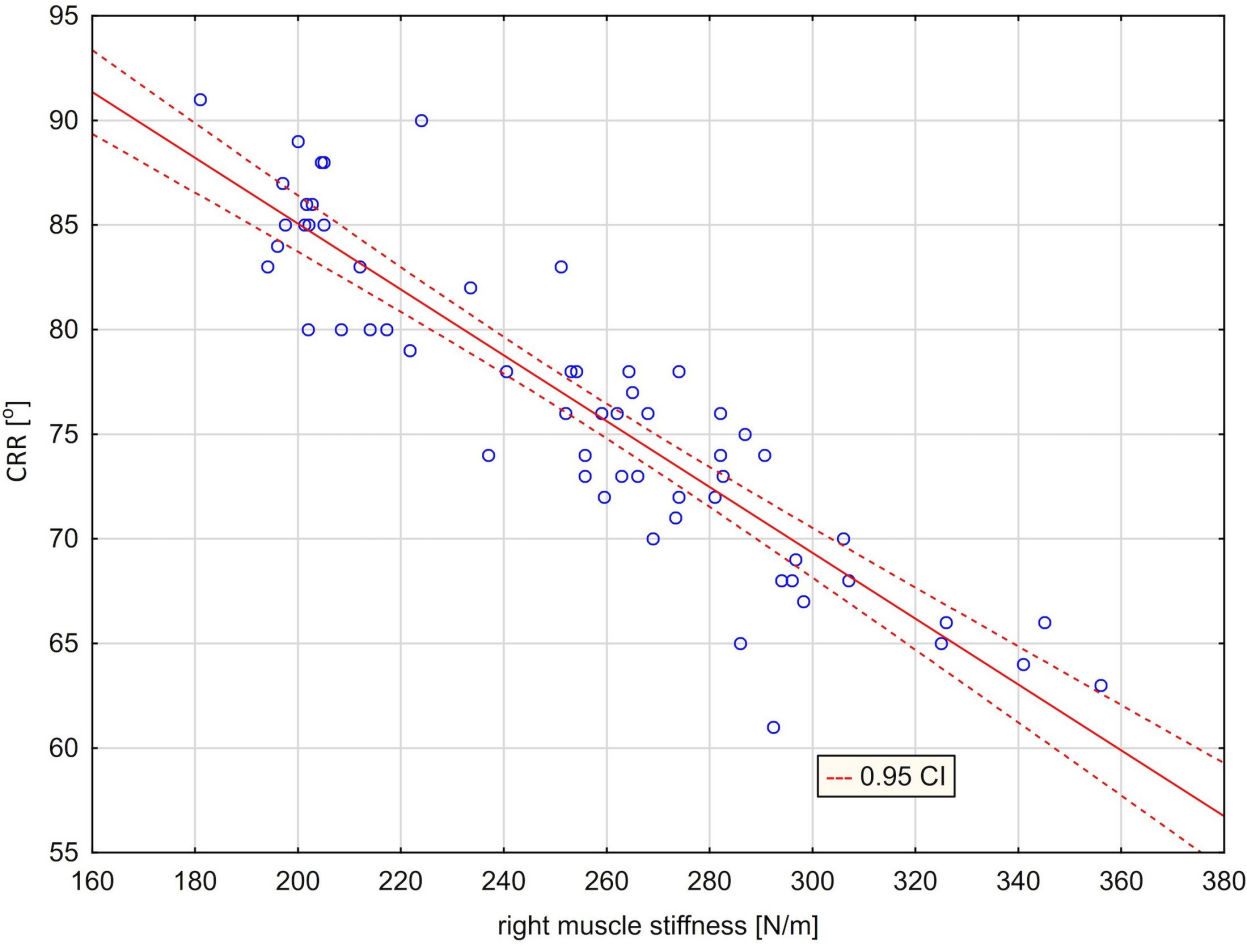
Scatter graph with fitted regression line [CRR = 116.53–0.1573*ST(R)] ‐ relationship between cervical right rotation [CRR] and right trapezius stiffness [ST(R)].

**Fig 5:**
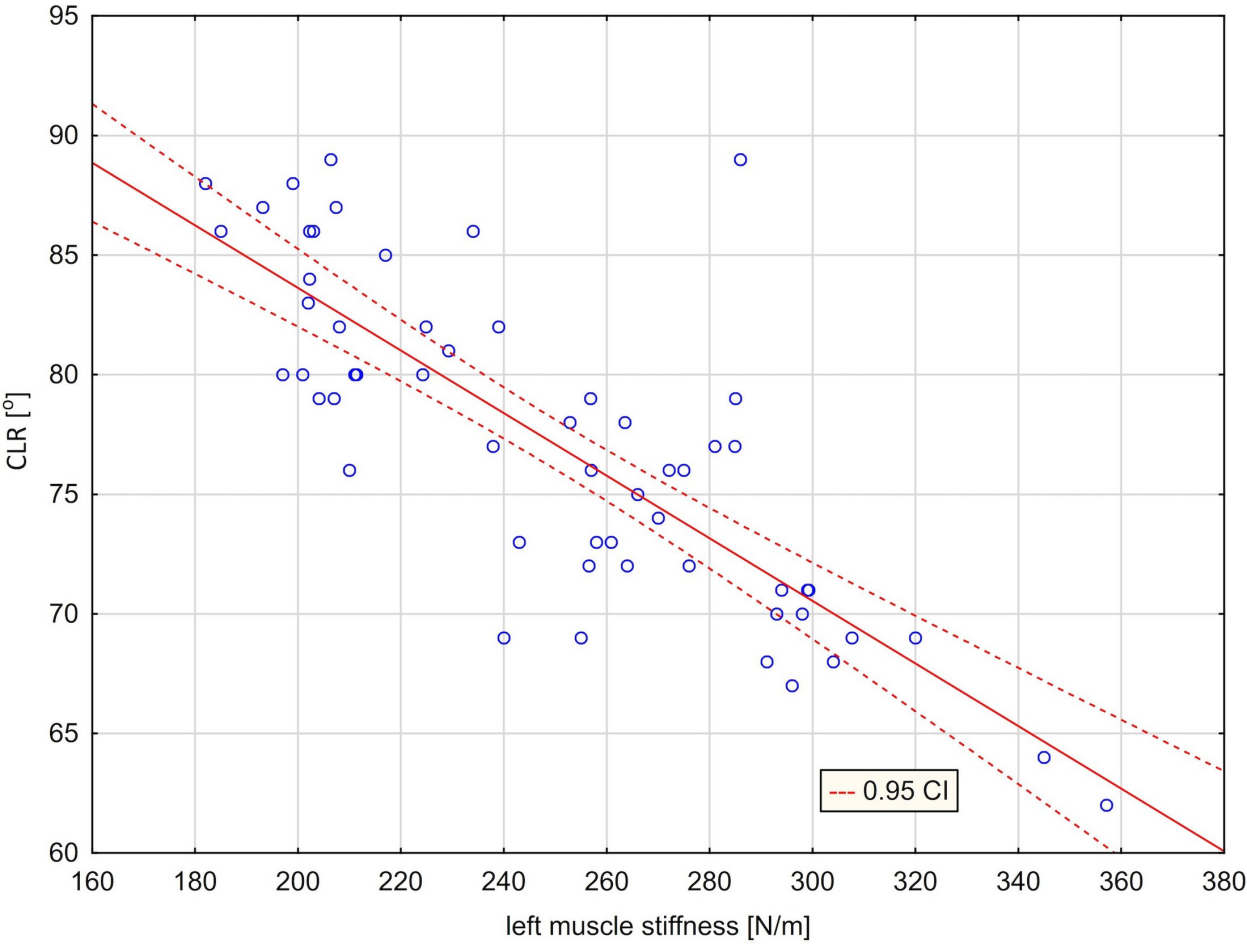
Scatter graph with fitted regression line [CLR = 109.80–0.1309*ST(L)] ‐ relationship between cervical left rotation [CLR] and left trapezius stiffness [ST(L)].

**Fig 6:**
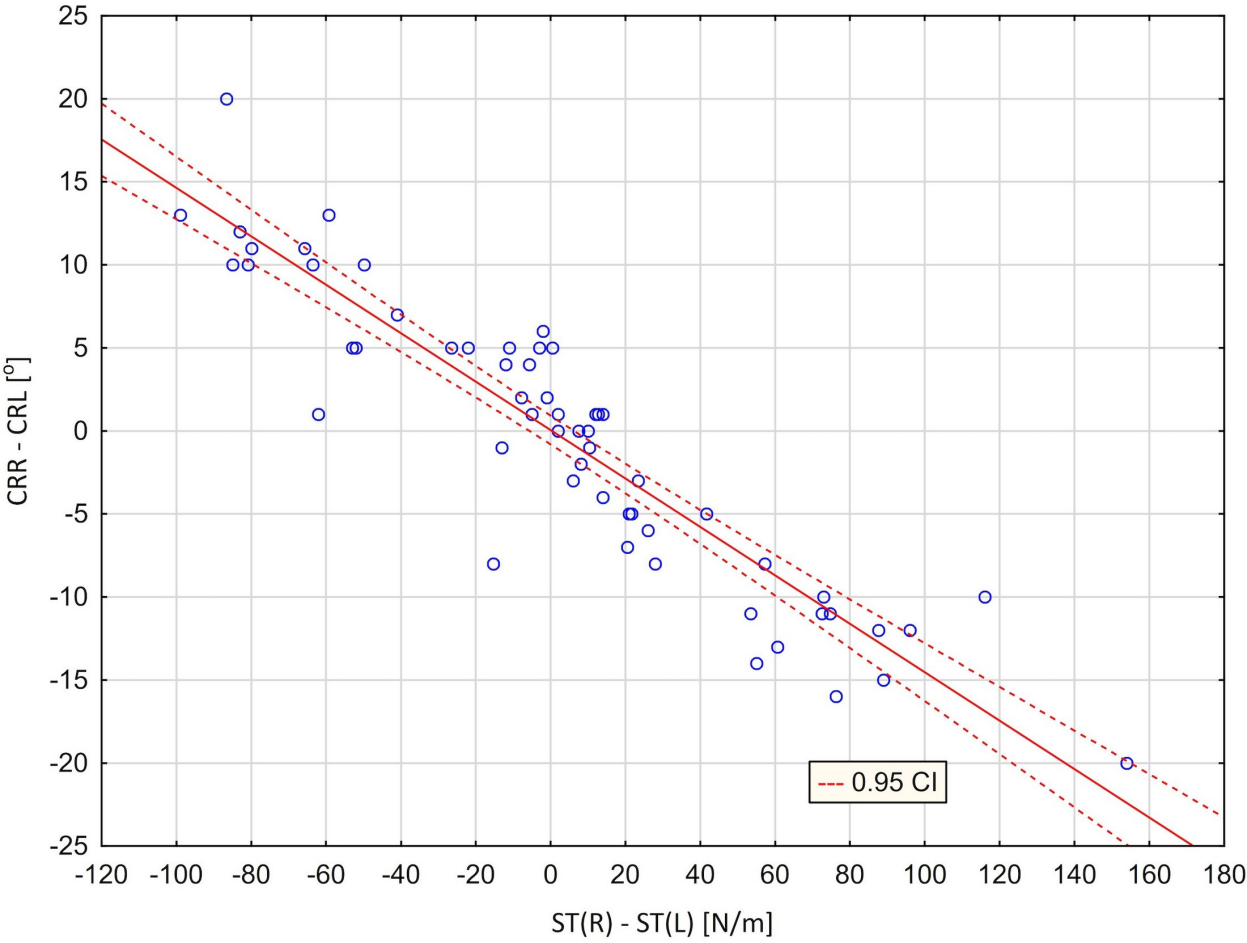
Scatter graph with fitted regression line [CLR–CLR] = 0.05427–0.1458*[ST(R)–ST(L)] ‐ relationship between the difference in right and left cervical rotation [CLR–CRR] and the difference in the stiffness of right and left trapezius muscles [ST(R)–ST(L)].

As a result of these analyzes, two regression lines were obtained showing a relationship between the stiffness of the right muscle and the extent of rotation to the right (Fig 4) and a relationship between the stiffness of the left muscle and the amount of left rotation (Fig 5). Figs 4 and 5 illustrating the presented models confirm a very good fit of the regression lines (marked with a solid line) to the actual data. The graphs also show the curves (marked with a dashed line), defining 95% confidence intervals for the expected values ​​of the modeled dependent variable. A regression analysis was also performed between the difference in the stiffness of the right and left muscles and the difference in the amount of right and left rotation (Table 10). A regression model that takes into account the independent variable [ST (R) ‐ ST (L)] explains more than 85% of the variance in the variable [CRR ‐ CLR]. The average difference between the actual values of the dependent variable and the values predicted by the model was 3.34°. The high value of the F statistic (332.28) and the corresponding p (p <0.001) probability level confirm the statistical significance of the linear model. The value of the t-statistic and the corresponding p-probability level confirm that the slope significantly differs from zero (Table 10). On the other hand, the y-intercept in the model does not differ significantly from zero (this means that the regression line runs very close to the center of the coordinate system) (Fig 6).

**Table 10:** Results of multiple regression analysis for the dependent variable CRR ‐ CLR and the independent variable ST (R) ‐ ST (L).

N = 60	Summary of the regression of the dependent variable: CRR–CLR R = 0.9227 R^2^ = 0.8513 F (1.58) = 332.28 p<0.0000 Standard error of estimation: 3.3378			
	b*	b	t (58)	p
y-intercept		0.054	0.126	0.9006
ST(R)–ST(L)	-0.9227	-0.146	-18.229	0.0000

R–multiple correlation coefficient, R^2^ –multiple determination coefficient, r–correlation coefficient, b–slope.

**Fig 7:**
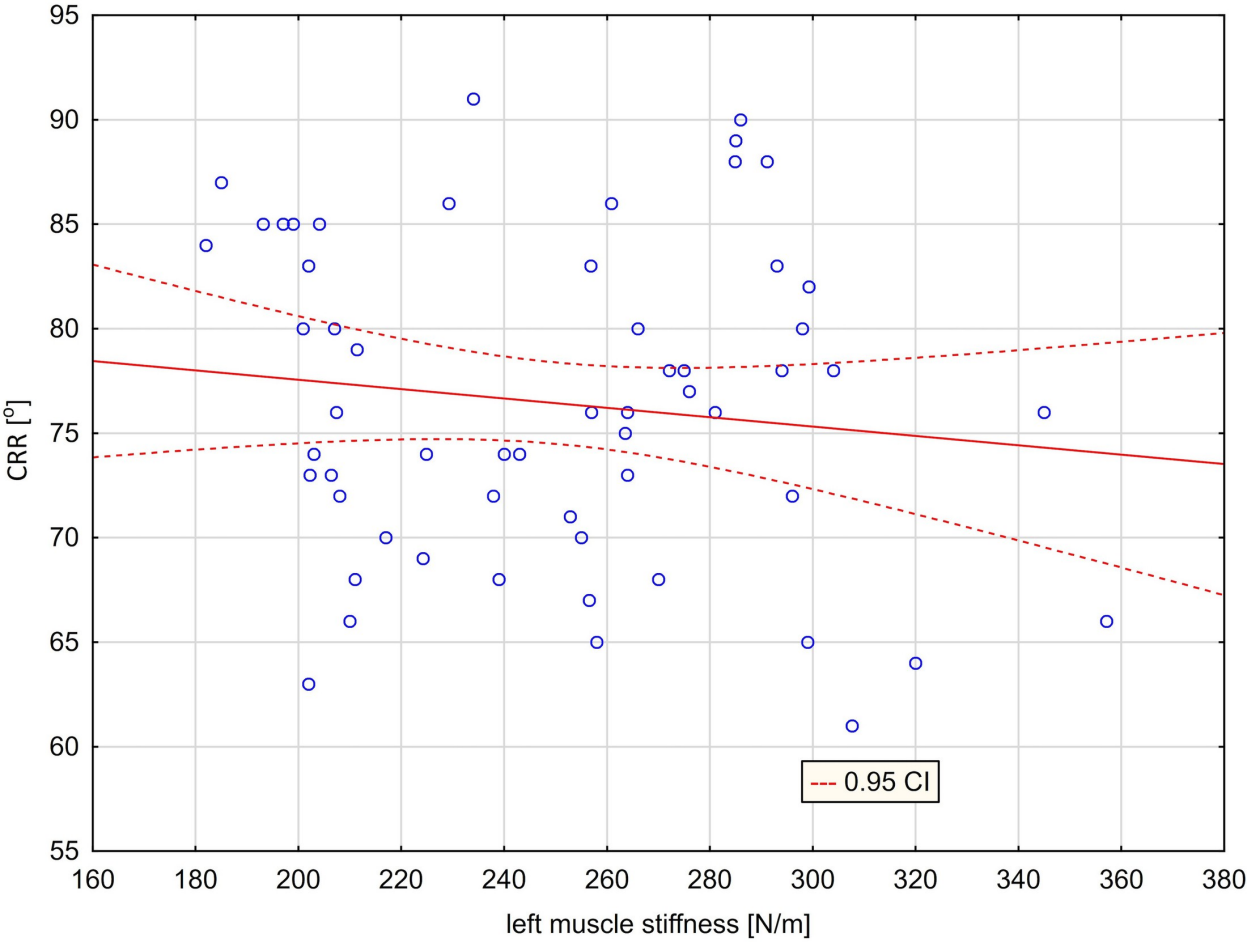
Scatter graph with mismatched regression line [CRR = 82.047–0.0224*ST(L), r = -0.1262] ‐ relationship between cervical right rotation [CRR] and left trapezius stiffness [ST(L)].

**Fig 8:**
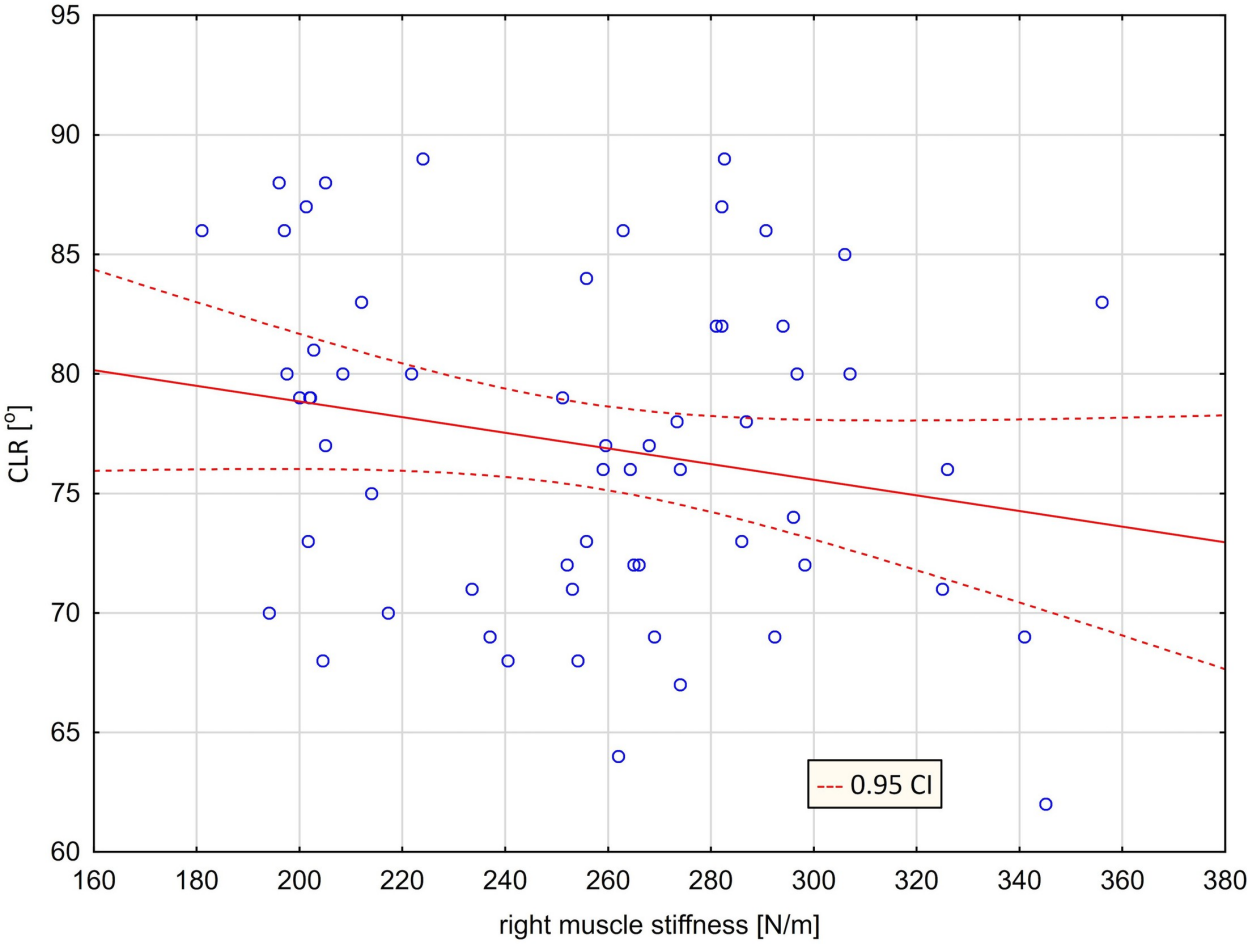
Scatter graph with mismatched regression line [CLR = 85.390–0.0327*ST(R), r = -0.2080] ‐ relationship between cervical left rotation [CLR] and right trapezius stiffness [ST (R)].

Figs 7 and 8 contain graphs showing the lack of relationship between left muscle stiffness and right rotation (Fig 7) and between right muscle stiffness and left rotation (Fig 8).

The results of multiple regression with the progressive stepwise method are confirmed in Table 11 presenting Pearson’s linear correlation coefficients. They were calculated to determine the strength of the relationship between the stiffness of the right and left trapezius muscles and the extent of right and left rotational motion. The extent of rotational movement to the right (right rotation) strongly correlates with the stiffness of the muscle on the right (r = -0.9069), and the rotation to the left correlates strongly with the stiffness of the muscle on the left (r = -0.8120). The calculated correlation coefficients are negative and significantly different from zero (p≤0.01) (Table 11). In the group of all subjects, there was no significant correlation between right rotation and left muscle stiffness and left rotation with right muscle stiffness (Table 11). For the next variable, which is the value of the difference in rotation to the right and left (CRR–CLR), the correlation with the variable which is the difference in the stiffness of the right and left trapezius muscle [ST(R)–ST(L)] is very strong and also negative (r = -0. 9227) (Table 11). A negative correlation coefficient means the presence of a type relationship: positive difference in rotation and negative difference in muscle stiffness. The greater the stiffness of the right muscle, the smaller the right rotation and the greater the stiffness of the left muscle, the smaller the left rotation.

**Table 11:** Correlation coefficients for the analysed variables, calculated jointly for all respondents (n = 60).

Variables		ST(R)	ST(L)
CRR	r	-0.9069	-0.1262
	p	p = 0.00**	p = 0.337
CLR	r	-0.2080	-0.8120
	p	p = 0.111	p = 0.000**
Variables		ST(R)–ST(L)	
CRR–CLR	r	-0.9227	
	p	p = 0.00**	

CRR ‐ cervical right rotation, CLR ‐ cervical left rotation, CRR–CLR ‐ difference of rotation to the right and left, ST(R)- stiffness of the right trapezius muscle, ST(L) ‐ stiffness of the left trapezius muscle, ST(R)–ST(L) ‐ difference between the stiffness of the right and left muscles trapezius ridge, r ‐ correlation coefficient, p ‐ test probability

** differences significant at the level of α≤0.01.

In summary, we obtained the following results:

1. Belonging to the experimental or control group did not significantly differentiate the stiffness of the upper trapezius muscle.
2. The side of limited rotation differentiated between right and left upper trapezius muscle stiffness only in the rotational movement asymmetry group.
3. A strong correlation was found between limited rotation of the cervical spine and stiffness of the upper trapezius muscle lying on the side of the rotational movement. This is evidenced by high correlation coefficients between the stiffness of the right trapezius muscle and restricted right rotation (r = -0.929) and between the stiffness of the left trapezius muscle and restricted left rotation (r = -0.855).
4. There was also a correlation between the difference in right and left rotation of the cervical spine and the difference in stiffness of the right and left upper trapezius muscle.

The results of the research indicate that increasing the stiffness of the right trapezius muscle may reduce the range of right rotation of the cervical spine and increasing the stiffness of the left trapezius muscle may reduce the range of left rotation.

## Discussion

### Explanation of the results

We found that the stiffness of the right and left trapezius muscles depended on the location of the muscle related to the range of rotational movement. In the experimental group (asymmetry of rotational movements ≥10°), the muscles on the side of limited (less) rotation of the cervical spine were significantly stiffer than the muscles on the side of greater rotation. The statistical analyses showed that limited rotation of the cervical spine is associated with increased stiffness of the trapezius muscle on the same side. It is probably related to the function of the upper trapezius muscle. In addition to affecting the shoulder girdle, this muscle rotates the cervical spine in the opposite direction [34]. Contraction of the upper right trapezius causes the cervical spine to rotate to the left. In this way, the muscle on the opposite side is stretched. Hence, increased stiffness of the upper trapezius, resulting from limited stretching of this muscle, may limit cervical spine rotation.

Stiffness of the upper trapezius muscle in the experimental group did not differ significantly from the stiffness of this muscle in the control group (not showing asymmetry in cervical spine rotation to the right and left). The lack of a statistical difference in stiffness between the studied groups may be due to the fact that belonging to a given group was not determined by the value of the range of motion, but by the asymmetry of rotational motion. In the control group, there was no significant variation in upper trapezius stiffness depending on the side of rotation. However, in the experimental group with asymmetry of rotational movements, there was a variation in the stiffness of the upper trapezius muscle depending on the side of limited rotation, which allowed us to reject the null hypothesis. No articles describing the relationship of the stiffness of the upper trapezius muscle to the asymmetry of rotational movements of the cervical spine in healthy subjects were found in the literature. It is assumed that in the case of disturbances in the spinal parameters of the spine, this statistical difference could appear. Liang et al. [35] showed a significant effect of the position of the cervico-thoracic spine on changes in the stiffness of the upper trapezius muscle. The authors noted that the increased neck flexion angle significantly increased the stiffness of the examined muscle (p <0.05). Incorrect position of the spine in the cervico-thoracic section increased the stiffness in the upper, middle and lower part of the trapezius muscle. The authors conclude that correct posture is important in maintaining the reduced stiffness of the back muscles and may contribute to reducing the incidence of spinal overload syndromes [35].

The correlation coefficients indicate that right rotation of the cervical spine correlates most strongly with the stiffness of the right trapezius muscle (especially with the one lying on the side of less rotation), and left rotation correlates most strongly with the stiffness of this muscle on the left side (especially on the side of less rotation). In the conducted study, greater stiffness of the trapezius muscle more strongly reduced rotation in the side on which the muscle lies than rotation in the direction opposite of that muscle. This is probably related to the stretching mechanism of this muscle rather than its contraction.

Muscles often play different roles, acting as force-generating motors and brakes that dissipate mechanical energy [36]. In many cases, these functions are not mutually exclusive, and a single muscle often performs a variety of mechanically different tasks. Muscles with different mechanical functions act on different areas of the force-length curve [37]. Muscle operative length can be influenced by potential trade-offs between force production and sarcomere stability, while passive stiffness acts to limit the working length of the muscle [38]. It is believed that there is a significant relationship between the motor function of the muscle and its mechanical properties [38]. Increasing the passive stiffness of a muscle may limit its maximum operational length during movement [38]. In our study, we observed a reduction in the rotation of the cervical spine in a given direction (right or left), which was significantly associated with an increase in the stiffness of the trapezius muscle lying on the same side as the restriction of movement. This is explained in the above theory, since the right upper trapezius muscle is stretched during the rotation of the cervical spine, and the left trapezius muscle in the left rotation.

### Influence of changes in muscle length on the stiffness parameter

There is evidence in the literature that changes in muscle length resulting from stretching or contraction significantly affect the stiffness parameter. Liu et al. [39] examined the stiffness of selected muscles of the lower limb in different settings of the knee joint and foot. In the case of passive stretching of the muscles (from the position of 50° plantar flexion of the foot to 25° dorsiflexion with the knee extended), there was a significant increase in stiffness [39]. For the gastrocnemius muscle, researchers found a 6-fold increase in this parameter in the area of the medial head and a 5-fold increase for the lateral head. The soleus muscle showed an almost 2-fold increase in stiffness, while in the proximal plantar fascia there was a 33% increase [39]. This phenomenon has also been confirmed in other muscles, including the tibialis anterior, tibialis posteriori, flexor digitorum longus and extensor digitorum longus [40].

Chang et al. [41] examined changes in the stiffness of the Achilles tendon depending on the foot position. The study was performed in a group of young, healthy participants. The authors noted that in the case of dorsiflexion of the foot (10°), the stiffness of the examined anatomical structure was clearly greater (p < 0.05) than in the case of setting the foot in the neutral position. This parameter increased by approximately 17%. The authors concluded that stretching the Achilles tendon increases its stiffness [41]. Tas and Salkin [42] confirmed the increase in Achilles tendon stiffness as a result of stretching. They noted a 40% increase in this parameter due to dorsiflexing the foot (10°). In turn, Aubry et al. [43] noted a 5-fold increase in Achilles tendon stiffness in the position of maximum dorsiflexion of the foot. The above scientific evidence shows that there is a relationship between muscle stretching position and increased stiffness. This applies to both muscles and tendons.

Several studies have shown a relationship between muscle contraction and an increase in muscle stiffness. In 2020, Yu et al. [44] reported changes in masseter muscle stiffness in a group of 20 healthy participants (10 women and 10 men). The mean stiffness of the examined muscle in the resting position was 369.5 N/m and it was 618.3 N/m at the maximum isometric contraction, for an approximately 67% increase in masseter muscle stiffness [44]. Kisilewicz et al. [45] investigated the effect of eccentric exercise on calf muscle stiffness in a group of 18 athletes. The authors noted a significant increase in stiffness (p < 0.001) due to the use of a single application of eccentric exercises (2 sets of 15 repetitions, 1 minute break between sets) [45]. The final measurement was performed immediately after the exercises. The authors noted a resting calf muscle stiffness of 323.5 ± 62.9 N/m and an increase in this parameter due to eccentric exercises to 392.7 ± 104.1 N/m [39]. Kelly et al. [46] assessed changes in stiffness depending on the strength of contraction (resting, 40% maximum voluntary isometric contraction [MVIC] and 80% MVIC) in the area of several muscles (infraspinatus, erector spinae and gastrocnemius). The authors noted an overall increase in stiffness depending on the strength of the muscle contraction. In the case of the infraspinatus muscle, the examined parameter increased by 46% at 40% MVIC and by 54% at 80% MVIC relative to the relaxed state. Erector spinae were characterised by a 31% increase in stiffness at 40% MVIC and by a 38% increase in stiffness at 80% MVIC. When examining the gastrocnemius muscle, the authors found a 45% increase in stiffness at 40% MVIC and a 50% increase in stiffness at 80% MVIC [46]. The above studies indicate that there is a clear relationship between muscle contraction and an increase in muscle stiffness.

### Influence of stiffness on the locomotor system

Muscle stiffness is a biomechanical property of a tissue that represents the resistance of a muscle (tissue) to contraction or a deformation force [4]. Increased stiffness affects the function of a muscle as well as adjacent structures. It is believed that increased stiffness means greater effort from the antagonist is required to stretch the muscle. This leads to an increase in the energy cost of the movement, which is less economical for the locomotor system [4].

Some studies suggest that passive muscle stiffness may also have positive practical effects. Greater passive muscle stiffness (for a given joint angle) can contribute to the rapid build-up of strength [47]. This can be important in many sports and in the training process of players. More research is necessary in terms of the influence of increased stiffness and positive practical functional advantages.

There are reports of a relationship between increased muscle stiffness and pain in the head and cervical spine. Park et al. [48] examined the biophysical parameters of the suboccipital muscles and the upper trapezius in a group of 40 participants, half of whom had a cervicogenic headache. The authors noticed a markedly greater tonus and stiffness of the examined muscles in the experimental group compared with the control group (asymptomatic people). Suboccipital muscles were 20% stiffer, while the upper trapezius was 17% stiffer [48]. The authors suggested that their results should be taken into account in cervicogenic headache therapy [48]. Tas et al. [49] reached similar conclusions in a study focussing at neck muscle stiffness in patients with chronic neck pain. The authors noted significantly higher values of this parameter in relation to the upper trapezius, levator scapulae and sternocleidomastoid in patients with chronic neck pain compared to the control asymptomatic group [49].

Studies conducted on a group of people with chronic low back pain have also shown a relationship between dysfunction of the locomotor system and increased stiffness of soft tissues. Wu et al. [50] assessed the biophysical parameters of lumbar extensor myofascia in a group of elderly people with chronic low back pain. In the experimental group, the average stiffness of the dorsal extensor was 320 N/m, while in the control group it was 277 N/m [50]. The authors concluded that the mean stiffness of the dorsal extensor muscles was significantly greater in patients with chronic low back pain than in healthy controls. There was also a positive correlation between the level of perceived pain and the stiffness of the examined muscles [50]. Andonian et al. [51] also noted a markedly greater (p < 0.021) stiffness in patients with ankylosing spondylitis compared with the asymptomatic control group. The study concerned lumbar myofascia and the age range of the participants was 18–46 years [51]. The data from the study showed that people with ankylosing spondylitis much more often (p < 0.012) had lumbar myofascial stiffness greater than 250 N/m [51]. The above scientific reports indicate a relationship between disorders of the lumbar spine and increased stiffness of the soft tissues in this area.

Park et al. [52] noted an improvement in subjective, functional and biophysical parameters after posteroanterior mobilisation in a patient with ankylosing spondylitis. The authors noted a 2–4° increase in mobility [52]. Improvement in mobility in the sagittal plane was not statistically significant, but it was already present in the frontal plane. There was also a reduction in muscle stiffness in the cervical spine (the measurement was performed 2 cm laterally from the C4 spinous process) as a result of the applied mobilisation. The authors noted a 77 N/m decrease (20%) in this parameter. The treatment effect lasted for 5 days [52]. Although the study was only a case study, it shows a relationship between stiffness and mobility in the cervical spine area.

There have also been studies suggesting the need to reduce muscle stiffness as a form of prophylaxis and protection of the musculoskeletal system. Zhou et al. [53] conducted a study related to the evaluation of calf muscle stiffness after the use of static stretching. The authors indicated that greater stiffness of the Achilles tendon and gastrocnemius muscles is a risk factor associated with the occurrence of tendinitis. The authors suggested that heterogeneous behaviour is present within the gastrocnemius muscle and the Achilles tendon, and this heterogeneity is reduced after static stretching. They concluded that stretching can effectively increase muscle flexibility, resulting in better protection of the locomotor system [53].

### Study limitations and future research directions

This study has several limitations. First, the observation was carried out on relatively small groups, which unfortunately was dictated by the concern for homogeneity of the study group. However, calculating the required sample size gave us acquiescence in the analyses and confidence that our results with a certain confidence level and assumed maximum error would estimate the true results in the population. Another limitation was the assumption of 10° for asymmetric right and left rotational movements. A larger difference between right and left rotation of the cervical spine between the study groups could indicate greater differences in the stiffness studied. The asymptomatic group is also a limitation to the study. Examining patients with pronounced cervical spine dysfunctions could allow more pronounced differences in stiffness to be captured in the intergroup analysis. A study assessing the stiffness of the upper trapezius muscle in the stretched position could also provide valuable information. It should be noted here that conducting such a study would be extremely difficult to carry out. It seems impossible to keep the active angular range of active rotation constant when performing stiffness measurements with MyotonPRO. We believe that in order to conduct such a study, the use of other research equipment should be considered.

In future studies, we plan to investigate the stiffness of the upper trapezius muscle in various ranges of mobility of the cervical spine. Evaluation of this parameter in groups of patients characterised by various dysfunctions of the musculoskeletal system should provide valuable data. Investigating the stiffness of several synergistic and antagonistic muscle groups while performing a given function could also be of scientific value.

## Conclusions

Based on the obtained results, we drew the following conclusions:

1. There is a relationship between the stiffness of the right and left upper trapezius muscles and the range of right and left rotational motion of the cervical spine.
2. Stiffness of the upper trapezius correlates more strongly with rotation to the side on which the muscle lies than to the opposite side (stiffness of the right upper trapezius correlates more strongly with rotation to the right, and of the left with rotation to the left).
3. Increased stiffness of the upper trapezius muscle on the side of limited cervical spine rotation is likely to be determined by the muscle fibre stretching mechanism.
